# Single Extracellular Vesicle Profiling to Define Brain Specific Traumatic Brain Injury Induced Neuro‐Inflammation

**DOI:** 10.1002/smtd.202401931

**Published:** 2025-05-19

**Authors:** Zhen Zhang, Richard J Lobb, Rebecca E Lane, Xuan Vinh To, Xueming Niu, Fiach Antaw, Giovanni Pietrogrande, Craig Winter, Alain Wuethrich, Fatima Nasrallah, Matt Trau

**Affiliations:** ^1^ Centre for Personalized Nanomedicine Australian Institute for Bioengineering and Nanotechnology (AIBN) The University of Queensland Saint Lucia QLD 4067 Australia; ^2^ The Queensland Brain Institute The University of Queensland Saint Lucia QLD 4067 Australia; ^3^ Australian Institute for Bioengineering and Nanotechnology The University of Queensland Saint Lucia QLD 4067 Australia; ^4^ Department of Neurosurgery Royal Brisbane and Women's Hospital Herston QLD 4006 Australia; ^5^ Faculty of Medicine The University of Queensland Saint Lucia QLD 4067 Australia; ^6^ School of Mechanical Medical and Process Engineering Queensland University of Technology Brisbane City QLD 4000 Australia; ^7^ School of Chemistry and Molecular Biosciences The University of Queensland Saint Lucia QLD 4067 Australia

**Keywords:** cytokine, extracellular vesicle, liquid biopsy, neuroinflammation, TBI

## Abstract

Traumatic Brain Injury (TBI) triggers secondary molecular processes that contribute to mortality and morbidity. Neuroinflammation is a key factor affecting patient outcomes both acutely and chronically. Traditional diagnostic tools, such as computed tomography imaging and the Glasgow Coma Scale, are limited in detecting molecular changes, particularly related to neuroinflammation. Small extracellular vesicles (sEVs) are cell‐specific vesicles that enable cell‐to‐cell communication and are involved in TBI pathology. In this study, brain‐specific sEVs are isolated by targeting brain‐associated markers, sodium/potassium‐transporting ATPase subunit beta‐2 (ATP1B2) and excitatory amino acid transporter 2 (EAAT2), and employed surface‐enhanced Raman spectroscopy to profile inflammation‐associated cytokine chemokine (C‐C motif) ligand 2 (CCL2) bound to single sEV, allowing for blood‐based monitoring of neuroinflammation. This approach enabled the direct assessment of neuroinflammation in both human TBI samples and a controlled cortical injury in a rat model. This study found elevated brain‐specific sEVs with enhanced CCL2 in TBI samples compared to non‐TBI cohorts. The results suggest that the TBI diagnostic platform can detect an increased level of brain‐specific sEVs carrying neuroinflammatory signals in TBI clinical samples with high specificity and sensitivity, offering potential as a precise diagnostic tool for TBI diagnosis.

## Graphical abstract

A highly innovative and implementable diagnostic tool for profiling microenvironmental perturbations in TBI patients by analyzing sEVs derived from glial cells circulating in the blood. This device provides a window into the injured brain and identifies neuroinflammatory signals, potentially serving as a platform for detecting highly specific brain biomarkers following TBI.

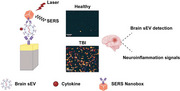

## 1 Introduction

Traumatic brain injury (TBI) poses a significant global health burden, with an age‐standardized incidence rate of 346 cases per 100 000 individuals worldwide.^[^ ^1^ ^]^ Patient outcome is a consequence of both the initial impact, causing structural damage to the brain, such as contusions, diffuse axonal injury, intracerebral hematoma, and intracranial vascular injuries, and the subsequent development of secondary molecular mechanisms triggered by the initial injury. These processes include, but are not limited to, lipid peroxidation, glutamate excitotoxicity, hypoxia‐ischemia, cytotoxic and vasogenic oedema, blood‐brain barrier (BBB) dysfunction, and neuro‐inflammation, ultimately leading to complex disruptions in neurological and psychological functions.

The initial severity of a TBI is currently assessed using clinical history, examination, and imaging findings. The Glasgow Coma Scale (GCS) is a primary diagnostic tool that stratifies head injuries into mild (GCS 13–15), moderate (9‐12), and severe (3‐8) categories based on the patient's level of consciousness.^[^ ^2^, ^3^, ^4^ ^]^ Furthermore, computed tomography (CT) scans are the primary imaging technology for acute TBI, defining structural damage to guide initial patient management, e.g., neurosurgery for evacuation of an extradural hematoma, intracranial pressure monitoring, or simply close observation.^[^ ^4^ ^]^ Magnetic resonance imaging (MRI) scans may also be used in certain situations to better define the initial structural injury. However, none of these diagnostic methods can accurately capture the intricacies and evolution of TBI pathology at the molecular level.^[^ ^5^ ^]^ Critically, mild TBI often presents with limited or non‐specific symptoms and is frequently overlooked by patients, despite its potential for long‐term neurological consequences.^[^ ^6^, ^7^ ^]^ Hence, there is a timely need for other diagnostic tests that may allow us to understand in greater detail the secondary processes that ultimately influence patient outcomes.

TBI‐related disruption of the BBB results in the potential movement of soluble factors from the brain parenchyma into the vascular compartment, such that brain‐specific proteins can be measured with a peripheral blood test.^[^ ^8^ ^]^ Due to BBB leakiness following TBI, several blood markers reflective of biological changes have been identified in peripheral blood, including glial fibrillary acidic protein (GFAP), neuronal‐specific enolase (NSE), ubiquitin C‐terminal hydrolase‐L1 (UCH‐L1), and neurofilament light (NfL).^[^ ^9^, ^10^ ^]^ However, the utility of these markers for liquid biopsy testing in TBI is limited, as current analysis focuses on bulk analyte concentrations in blood, rather than specifically measuring microenvironmental perturbations around neurons, glia cells, and immune cells in the brain parenchyma.^[^ ^11^ ^]^ Given the heterogeneous nature of the TBI population, accurately defining improvement in outcome following the use of a therapeutic trial, for example, can be extremely problematic. Alterations in the temporal profile of a reliable and accurate biomarker provide the possibility of a surrogate marker of the amelioration of harmful molecular changes following a novel intervention.

Neuroinflammation is a key factor that actively influences patient outcomes. Many studies have shown that neuroinflammation is associated with both acute and chronic TBI and is a central component of the injury.^[^ ^12^, ^13^ ^]^ The impact of neuroinflammation following TBI may persist for up to 17 years post‐injury, contributing to progressive impairments in patients’ physical, cognitive, and social performance.^[^ ^14^, ^15^ ^]^ However, current peripheral biomarkers that are considered to reflect neuroinflammation may not universally represent isolated changes in the brain. TBI patients will often have other injuries, either soft tissue, visceral, or skeletal, and these will induce a systemic inflammatory response. In addition, over the course of the patient's intensive care unit stay, other medical issues will cause generalized inflammatory responses, e.g. an infection, thrombo‐embolic complications, and iatrogenic elevations following surgery. Hence, the need for an assay able to definitively measure brain cell‐specific biomarkers, either glial or neuronal.

Small extracellular vesicles (sEVs) are nano‐sized vesicles (50–150 nm) released from cells, which represent the cell‐of‐origin and the local microenvironment they are derived from.^[^ ^16^, ^17^, ^18^ ^]^ Isolating brain‐specific sEVs enables the direct assessment of neurobiological changes associated with TBI. Previously, cytokines such as chemokine (C‐C motif) ligand 2 (CCL2) have been shown to bind to the glycosaminoglycan side chains of proteoglycans on the surface of sEVs.^[^ ^19^ ^]^ This unique interaction can be utilized to specifically interrogate the neuroinflammatory microenvironment by profiling cytokine levels on sEVs released from the brain. However, analyzing brain sEVs is challenging due to a lack of highly sensitive analytical tools capable of detecting trace sEVs released from the brain into the peripheral circulation.

To address this challenge, we pioneered a digital decoding technique for single sEVs analysis.^[^ ^20^ ^]^ Using this approach, we captured brain‐specific sEVs to measure proteins that reflect local microenvironmental neuroinflammation at the site of a TBI. In this study, we successfully isolated brain‐specific sEVs using a unique brain signature based on transmembrane proteins, specifically sodium/potassium‐transporting ATPase subunit beta‐2 (ATP1B2) and excitatory amino acid transporter 2 (EAAT2), known for their high expression in normal central nervous system (CNS) tissue.^[^ ^21^, ^22^, ^23^ ^]^ Elevated levels of CCL2 have been found to correlate with post‐traumatic inflammation.^[^ ^24^, ^25^ ^]^ Therefore, our study analyzed CCL2 bound to brain‐specific sEVs as a potential marker for monitoring neuroinflammation. In a rat model of moderate‐severe TBI, we observed consistent elevation of brain‐specific sEVs that were positive for CCL2 up to 60 days post‐brain injury, signifying enduring neuroinflammation. Furthermore, our single sEVs platform detected a significant increase in CCL2 levels on brain sEVs in TBI patients compared to healthy individuals with high specificity and sensitivity. Overall, our TBI nanopillar chip demonstrates promise in detecting neuroinflammation by profiling cytokine levels on brain‐specific sEVs.

## 2 Experimental Section

### 2.1 Materials

Izon qsEV Original 70 nm columns were purchased from Izon Science. Amicon Ultra‐15 100 kDa MWCO columns and Amicon Ultra‐4 10 kDa MWCO columns were purchased from Merck. 96‐well U‐bottom low attachment plate, Matrigel, and 24‐well low attachment plate were purchased from Corning. Phosphate‐buffered saline (PBS), dithiobis (succinimidyl propionate) (DSP), Dimethyl sulfoxide (DMSO), bovine serum albumin (BSA), and Tween‐80 were purchased from Sigma–Aldrich. Antibodies used in our study include, anti‐ATP1B2 (PA526279, Thermo Fisher Scientific), anti‐EAAT2 (NOVNBP120136, Novus Biologicals) and anti‐CCL2 (505901, Biolegend), anti‐CD63 (H5C6, eBioscience), and anti‐CD81 (1D6, eBioscience).

### 2.2 Study Subjects and Participants

The TBI and control participants’ demographics have been previously reported^[^ ^26^, ^27^ ^]^ and approved by the Institutional Human Research Ethics Committee (approval number HREC/16/QRBW/604). Twenty‐seven patients were recruited between 2009 – 2021 with 19 controls. Exclusion criteria included: age < 18 or > 80, previous neurodegenerative disease, or significant mental health disorders. All patients had positive findings on their initial CT scan or met the National Institute of Neurological Disorders and Stroke (NINDS)^[^ ^28^ ^]^ or American Congress of Rehabilitation Medicine (ACRM)^[^ ^29^ ^]^ definition of TBI, which included a documented loss of consciousness or post‐traumatic amnesia following a head injury. Written informed consent was obtained from either the patient or the substitute decision maker. It was not possible to gain immediate blood tests given the often confused nature of the patient cohort, the delay in gaining consent from the next of kin, and involvement in other TBI research MRI trials. Blood samples were collected into ethylenediaminetetraacetic acid (EDTA) collection tubes and centrifuged at 1600 g for 10 min, with the plasma fraction collected for storage at −80 °C until analysis. A group of 19 demographically matched healthy controls was recruited for a donation of blood samples. Selection criteria for the controls included absence of neurodegenerative disease, mental health disorders, or TBI within the preceding 12 months. The clinical demographics and sample data are listed in Tables S1 and S2 (Supporting Information).

### 2.3 Controlled Cortical Impact Traumatic Brain Injury Model

The moderate‐severe traumatic brain injury rat model and blood sampling have been described in earlier publications^[^ ^30^, ^31^ ^]^ and were reproduced here for completeness. This part of the study was approved by the Animal Research Ethics Committee (AEC) of the University of Queensland (IRB number: QBI/036/16/MAIC). Nine Sprague‐Dawley male rats (8–10 weeks old, 300–340 g) were obtained from the Animal Resource Centre (ARC, Western Australia) and housed under conventional laboratory conditions with a 12‐h light‐dark cycle; food and water were available ad libitum. Two groups were used: sham surgery (*n* = 5) and TBI (*n* = 4), and the rats were randomly assigned to each group. Tail vein venipuncture was used for blood sampling at days 1, 3, 7, and 60 post‐sham or TBI procedure; plasma fractions were separated and frozen for later analysis.

We used the controlled cortical impact (CCI) head injury model. Rats were anesthetized with isoflurane (5% for induction, 1–2% for maintenance) in a 40:60 O2: medical air gas mixture delivered a flow rate of 2 L min^−1^. The rats were transferred to a stereotaxic frame, and the brain was exposed through a 5 mm craniotomy window centered at 2.5 mm posterior to the Bregma and 3 mm right lateral to the sagittal suture. A CCI was delivered to the exposed dura mater by a pneumatically driven impactor (TBI 0310, Precision System and Instrumentation, USA) with a cylindrical 4 mm diameter tip, impact velocity = 5 m s^−1^, penetration depth = 2 mm, and dwell time = 200 ms. For sham animals, the craniotomy was performed as above, but no CCI head injury was delivered. After the impact or sham procedure was performed, the bone flap was replaced over the craniotomy window, and the scalp was sutured. Total time under anesthesia was under 20 min. Animals were then placed on a heated surface for monitoring and recovery; they were returned to their home cages after they had recovered and appeared mobile and alert. Blood sampling was performed by tail vein venipuncture at day 1, 3, 7, and 60 post‐procedure, and the blood was collected into a 1.5 mL Eppendorf tube coated with 8 µL of 0.5 m EDTA. An additional volume of EDTA solution was added to blood samples to reach a final 5 mm concentration. Blood samples were centrifuged, and the plasma fraction extracted, which was then filtered by re‐centrifugation twice, first through glass wool, then through a 0.22‐micron filtration column. The filtered plasma samples were stored at −80 °C for further analysis.

### 2.4 Cell Culture

Undirected human brain organoids were prepared starting from a single cell suspension of the iPSC line A18945 as previously described^[^ ^32^ ^]^ with some modifications. Briefly, embryoid bodies (EBs) were generated by seeding 11 000 single cells in each well of a low attachment 96‐well U‐bottom low attachment plate (Corning) with 10 µm Y‐27632 for two days, and then replaced by EB medium for 6 days, followed by Neural induction media in the same 96‐well plate. On day 11, EBs were transferred on sheets of parafilm, covered with 13 µL of 100% Matrigel (Corning), and incubated for 25 min at 37 °C for embedding. Matrigel embedded single EBs were then transferred to a 24‐well low attachment plate (Corning) in 1 mL of differentiation media per well, and replaced every three times a week for 100 days.

### 2.5 sEVs Purification

sEVs were purified from cell culture medium and plasma (human and rat) using size exclusion chromatography (SEC) as previously described.^[^ ^33^, ^34^ ^]^ Cell culture medium was centrifuged at 500 g for 10 min to remove cellular debris. The supernatant was then filtered with 0.22 µm filter to remove large particles before being concentrated to 500 µL with an Amicon Ultra‐15 100 kDa MWCO columns (Merck) at 3500 ×g for ≈45 min at 4 °C. Frozen plasma from rat and human were thawed rapidly at room temperature and centrifuged at 10,000 g for 10 min at 4 °C to remove any remaining cellular debris. Subsequently, 500 µL of concentrated cell culture media or plasma was overlaid on an Izon qsEV Original 70 nm columns (Izon Science) and eluted using filtered PBS (Sigma‐Aldrich). Following the void volume, the following 1.6 mL of sEVs enriched fractions were collected and concentrated to ≤ 50 µL utilizing Amicon Ultra‐4 10 kDa MWCO columns (Merck) at 3500 ×g for ≈45 min at 4 °C. The resulting concentrated sEVs isolates were then stored at −80 °C before use.

### 2.6 sEVs Size and Concentration Measurements

The size and concentration of sEVs were measured using a nano flow cytometry (nanoFCM) (Figure S1, Supporting Information) equipped with 488 and 640 nm lasers. QC nanospheres, with a known concentration of 4.96 × 10^10^/mL were used to calibrate the instrument and for determining concentration at a 1 kPa sampling pressure. For size calibration, a standard working curve for scattering light intensity was established using a four modal silica nanoparticle mix (S16M‐Exo, 68–155 nm) at a 10 mW laser power, 10% size scattering (SS) decay, and a 1 kPa sampling pressure. For sEVs analysis, samples were diluted so that the number of events recorded was between 4000 and 8000/min, and measured at a 10 mW laser power, 10% SS decay, and a 1 kPa sampling pressure.

### 2.7 sEVs Immunofluorescent Staining

Approximately 1 × 10^9^ total sEVs particles were incubated with 3 µg of anti‐CD81 (1D6, eBioscience) in 110 µL of PBS, for 1 h at RT. Labeled sEVs were diluted with 400 µL of PBS and loaded on an Izon qsEV Original 70 nm columns (Izon Science). Following the void volume, the following 1.6 mL of sEVs enriched fractions were collected and concentrated to ≤50 µL with an Amicon Ultra‐4 10 kDa MWCO columns (Merck) at 3900 ×g for ≈20 min at 4 °C to ≈100 µL. Samples were analyzed with the nanoFCM equipped with 488 and 640 nm lasers. Data was analyzed using FlowJo version 10.10.0.

### 2.8 Transmission Electron Microscopy

Transmission electron microscopy (TEM) was used to visualize purified sEVs as previously described.^[^ ^33^ ^]^ Briefly, for TEM analysis, 2.5 µL of isolated sEVs (1 × 10^10^ particles/mL) were fixed with an equal volume of 2% glutaraldehyde for 30 min at room temperature. Fixed sEVs were then loaded on Formvar/carbon‐coated electron microscopic grids (Electron Microscopy Science) and incubated for 15 min. Following this, the grid was washed three times with 100 µL of Milli‐Q water. sEVs were negatively stained with 30 µL of 2% uranyl acetate (w v^−1^) for 5 min and observed using transmission electron microscopy (Hitachi HT7700) at 100 kV. All the TEM images of sEVs were shown in Figure 2A and Figure S1 (Supporting Information).

### 2.9 Surface Enhanced Raman Spectroscopy Nanoparticle Synthesis

Surface Enhanced Raman Spectroscopy (SERS) nanotags were prepared by functionalizing Au–Ag alloy nanoboxes with Raman reporters and antibodies as previously reported.^[^ ^35^ ^]^ Following this, 1 mL of nanobox solution was centrifuged at 800 × g for 15 min and resuspended in 200 µL of H_2_O. Subsequently, 10 µL of 1 mm Raman reporters (i.e., MBA, DTNB, and MMC) and 2 µL of 1 mm DSP (Sigma–Aldrich) in DMSO (Sigma–Aldrich) were incubated with the nanobox solution at 25 °C for 6 h. After removing free Raman reporters and DSP, 1 µg of anti‐CD63 (H5C6, eBioscience) or anti‐CD81 (1D6, eBioscience), anti‐APT1B2 (PA526279, Thermo Fisher Scientific), anti‐EAAT2 (NOVNBP120136, Novus Biologicals) and anti‐CCL2 (505901, Biolegend) were added into the nanobox solution and incubated overnight at 4 °C. Finally, the SERS nanotags were purified by centrifugation at 800 × g for 15 min and dissolved into 0.1% BSA (Sigma–Aldrich) for storage at 4 °C.

### 2.10 TBI Nanopillar Chip Fabrication

The TBI nanopillar chip measured 1 mm by 1 mm and contained ≈250 000 pillars. The pillars are 1 µm wide, 1 µm long, and 1 µm deep. Each pillar was separated by 1 µm from adjacent pillars. The TBI nanopillar chip was fabricated on a 4‐inch silicon wafer using a stepwise process of electron beam lithography, electron beam metal evaporation, and reactive ion etching, as reported previously.^[^ ^35^ ^]^

### 2.11 TBI Assay

Prior to use, the TBI nanopillar chips were washed with isopropanol, acetone, and water. The gold surface was activated by incubating 10 µL of 5 mm Dithiobis (succinimidyl propionate) (DSP, Sigma–Aldrich) for 30 min at room temperature. The chip was then washed with ethanol, water, and 1× PBS, before 10 µL of 10 µg mL^−1^ anti‐ATP1B2 and anti‐EAAT2 antibody in 1× PBS was incubated 2 h at room temperature. Then the chip was blocked by adding 10 µL of 5% BSA (Sigma–Aldrich) for 1 h at room temperature. Next, the chips were washed three times with 1% BSA in PBS. 10 µL of sEVs was added to the nanopillar chip (E7 particles from brain organoid samples and E8 particles from rat and human plasma samples), incubated for 30 min at room temperature, and washed three times with washing buffer (0.01% Tween‐80, 0.1% BSA, 1× PBS) (Sigma–Aldrich). Subsequently, 10 µL of SERS nanoboxes were incubated on the chip for 30 min at room temperature, washed three times with washing buffer, and stored at 4 °C prior to Raman mapping.

### 2.12 Raman Mapping and Data Analysis

Raman mapping was conducted using a Witec Alpha 300 R microscope equipped with an EMCCD camera, a 100 × objective (numerical aperture, NA = 0.9), and specific parameters: a He‐Ne laser (632.8 nm), laser power set at 35 mW, grating of 600 g mm^−1^, spectral resolution within the range of 1.390^−2^.114 cm^−1^, integration time of 0.01 s, scanning area size of 60 µm  ×  48 µm, scanning area resolution at 86 points per line, and 69 lines per image. To calibrate the system initially, the intensity of the Si peak at 520 cm^−1^ was measured using a Si wafer. Mapping of the pillar array began with the objective focused on the Si substrate, and then the 100 × objective was raised by 1 µm along the z‐axis to commence SERS mapping. A total of nine areas were scanned, covering a 60 µm × 48 µm area per pillar array, resulting in the scanning of a total of 6580 pillars.

### 2.13 Statistical Analysis

All statistical analyses were performed using GraphPad Prism (GraphPad Software v.10.1.2), and values are given either as mean ± SEM or SD. Two‐way ANOVA was used for multiple comparisons, and *P* values were adjusted using Šidák for multiple comparisons. When two groups were compared, significance was determined using an unpaired two‐tailed *t*‐test. *P* value threshold of <0.05 was considered statistically significant.

## 3 Results and Discussion

### 3.1 Principle of TBI Nanopillar Chip for Detection of TBI

**Figure 1:**
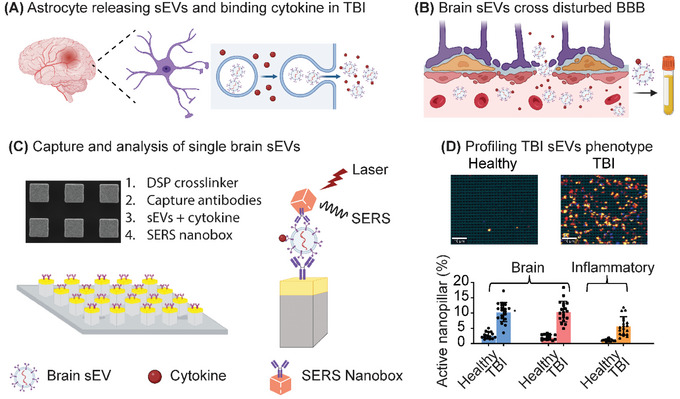
Single extracellular vesicle profiling to define brain specific TBI induced neuro‐inflammation. A) Astrocyte sEVs bind cytokines present in a neuroinflammatory microenvironment. B) sEVs cross the disrupted blood‐brain barrier (BBB) into peripheral blood and are subsequently isolated from plasma C) The single sEV readout platform captures individual sEVs, phenotypically barcoded with SERS nanobox reporters. The process includes: 1. activation of the gold surface with the crosslinker DSP, 2. immobilization of capture antibodies, 3. binding of sEVs, and 4. incubation with SERS nanobox reporters. D) BBB breakdown and neuroinflammatory changes are quantified in healthy controls and TBI patients by the amount of brain specific sEVs positive for CCL2.

At the site of a TBI injury, CCL2 is rapidly produced by astrocytes and plays a key role in guiding cell migration in the injured brain.^[^ ^36^ ^]^ CCL2 binds to the glycosaminoglycan side chains of proteoglycans on the surface of sEVs released by cells in the local microenvironment (**Figure** **1**A). We hypothesize that CCL2 positive brain sEVs cross into peripheral blood due to BBB breakdown and can be utilized to profile neuroinflammation in the brain (Figure 1B). These brain‐specific sEVs are captured on a nanopillar platform functionalized– anti‐ATP1B2 and anti‐EAAT2 for single sEV readout (Figure 1C; Figure S2A, Supporting Information). Silver/gold alloy SERS nanoboxes are used as analytical probes to profile the presence of ATP1B2, EAAT2, and CCL2 protein on brain sEVs (Figure 1C; Figure S2B, Supporting Information). As a result, BBB breakdown and neuroinflammatory changes in the brain can be identified in patients and used to diagnose TBI (Figure 1D).

### 3.2 Characterization of sEVs

**Figure 2:**
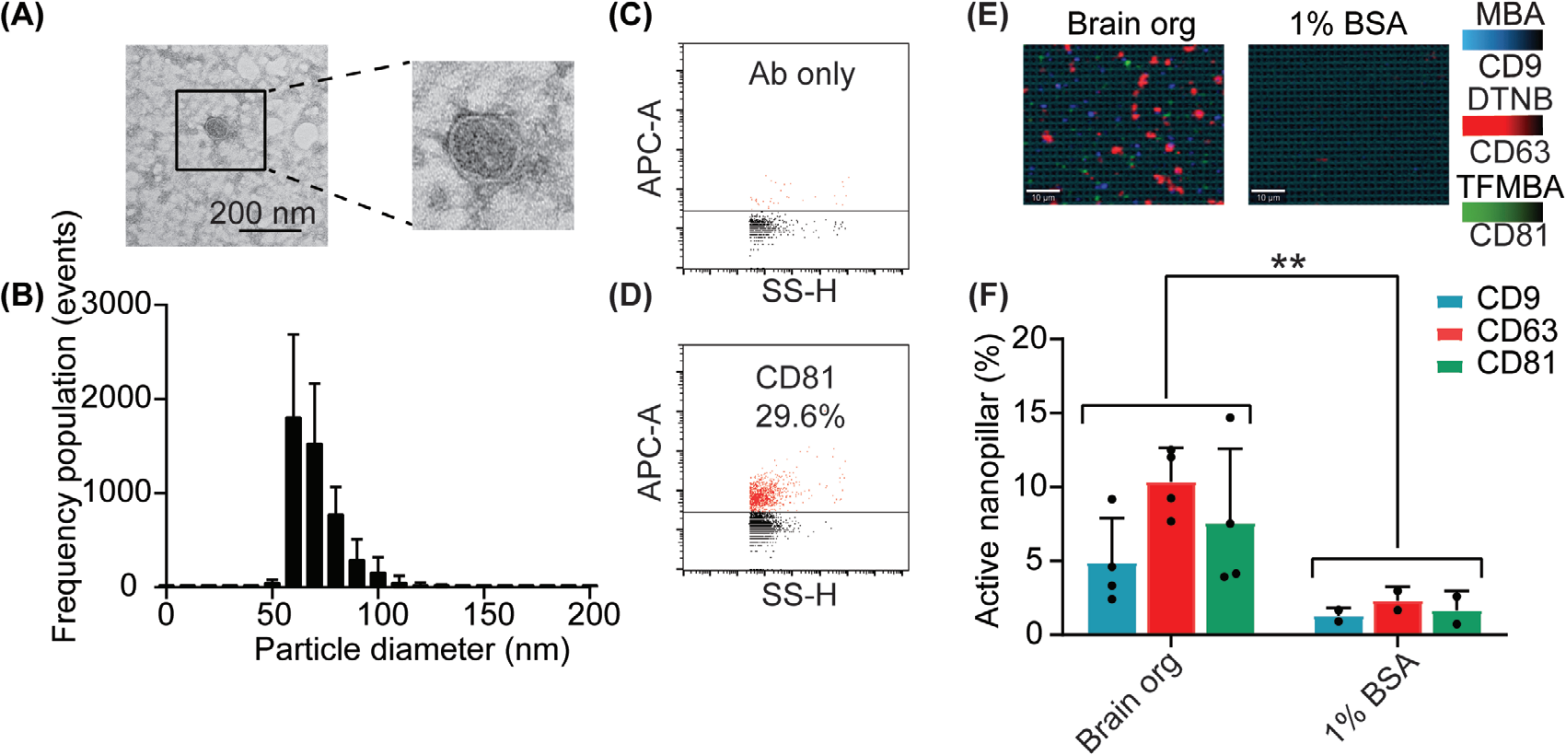
Brain‐specific sEV characterization. A) TEM image of brain organoid sEVs. B) Brain organoid (brain org) sEV characterization by NTA, demonstrating the size range of purified sEVs primarily between 50 and 100 nm. Nano flow cytometry of the sEV marker CD81: C) negative control (antibody only); D) the expression of tetraspanin marker CD81 on brain organoid sEVs. E) SERS signals of canonical tetraspanin markers CD9, CD63, and CD81, indicating the successful capture of brain sEVs using ATP1B2 and EAAT2 on the nanopillar. F) Quantification of nanopillars positive for the tetraspanin makers from sEVs isolated from human brain organoid cultures. Data are represented as mean ± SD. ^**^ *p* < 0.01.

Prior to the capturing and analyzing of brain specific sEVs from clinical samples, we first tested the capability of our nanopillar chip in capturing brain sEVs. sEVs were isolated from undirected human brain organoid cultures through an ultrafiltration and size exclusion chromatography (SEC) protocol we have previously optimized.^[^ ^34^ ^]^ The physical and biological properties of the organoid derived sEVs were characterized for morphology, size distribution, and expression of canonical tetraspanin (CD9, CD63, and CD81). TEM analysis confirmed that lipid bilayer vesicles were successfully purified (**Figure** **2**A), which a size distribution between 50–150 nm in diameter (60 nm mode) (Figure 2B). Using nano flow cytometry we detected 29.6% of CD81‐positive sEVs in brain organoid cultures (Figure 2C,D). We then demonstrated that ATP1B2‐ and EAAT2‐ positive sEVs from brain organoid cultures were captured on our nanopillar chip, using the canonical sEV tetraspanin proteins CD9, CD63, and CD81 as a readout (Figure 2E,F). We observed a significant difference in the expression of tetraspanin proteins in brain organoid sEVs compared to the negative control (Figure 2F). Overall, these results demonstrate the reliable purification and specific capture of sEVs on the TBI nanopillar chip.

### 3.3 Specificity of the SERS‐Based Nanopillar Chip

**Figure 3:**
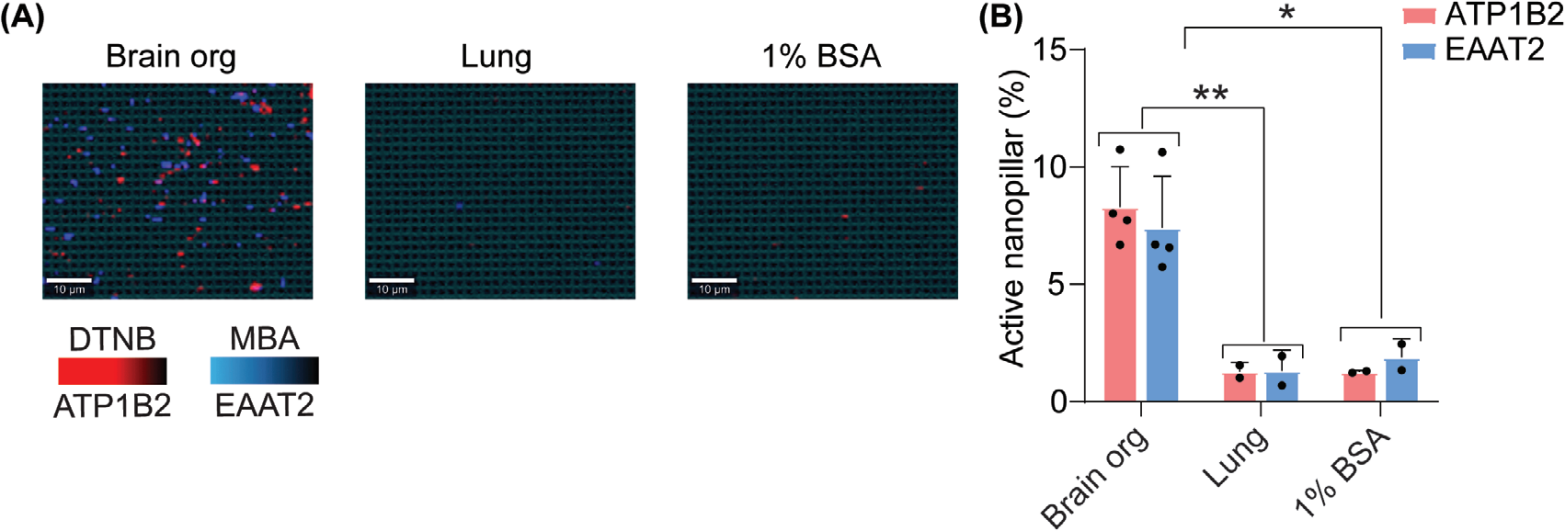
Brain sEVs marker validation. A) SERS images of sEVs from human brain organoids and control sEVs from lung cells (H1975). Red and blue dots indicate ATP1B2 and EAAT2 positive sEVs respectively. B) Quantification of nanopillars that are positive for our candidate brain specific markers ATP1B2 and EAAT2. Data are represented as mean ± SD. ^*^ *p* < 0.05, ^**^ *p* < 0.01.

Next, we aimed to validate the specificity of our approach in capturing sEVs derived from the brain using ATP1B2 and EAAT2. The RNA expression of ATP1B2 and EAAT2 was analyzed using the GTEx and Brain RNA‐seq databases. High levels of ATP1B2 and SLC1A2 (EAAT2) expression were observed in brain tissue and astrocytes compared to other tissues and brain cell types (Figure S3, Supporting Information). In addition, these two membrane proteins were previously validated in our study^[^ ^23^ ^]^ as being specifically expressed on sEVs derived from the CNS. To further test the specificity of these two markers in current study, we used sEVs from our positive control human organoid system, and a negative sEV control derived from lung epithelial cells using the H1975 adenocarcinoma cell line. Using a dual capture and readout of ATP1B2 and EAAT2 positive sEVs, we confirmed that only sEVs derived from our human brain organoid model were captured, with no signal detected from our lung‐derived sEVs (**Figure** **3**). Based on the validations from previous and current studies, we suggest that our candidate markers, ATP1B2 and EAAT2, are specific for capturing brain specific sEVs.

### 3.4 In Vivo Monitoring of Neural Inflammation in Response to a TBI

**Figure 4:**
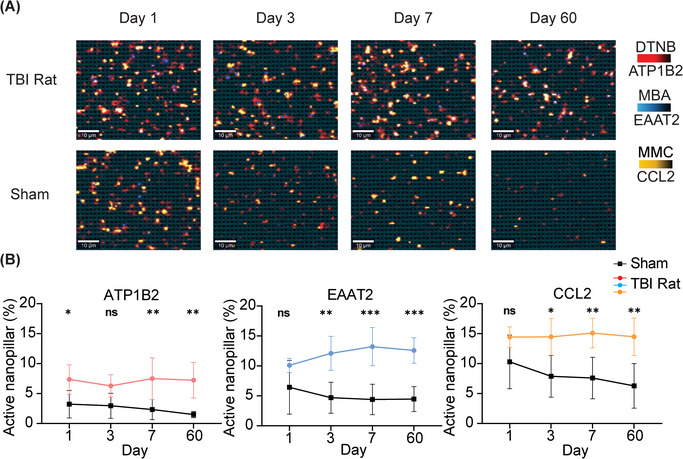
Specific profiling of brain sEVs in rats after a controlled cortical impact. A) Representative SERS mapping images of the sEVs from rats with a TBI and control sham rats. B) Corresponding sEV molecular profiles from panel A demonstrating elevated ATP1B2, EAAT2, and CCL2 in response to a TBI are sustained for 60 days post‐injury. *n* = 4 (TBI); *n* = 5 (Sham), data are represented as mean ± SD. ^*^ *p* < 0.05, ^**^ *p* < 0.01, ^***^ *p* < 0.001.

After evaluating the nanopillar chip's performance in terms of specificity, we further investigated its capability to monitor neuroinflammation using brain specific sEVs in vivo in a rat controlled cortical impact (CCI) model. CCI injury was induced after craniotomy using a pneumatically driven impactor, which we have described in detail previously.^[^ ^37^ ^]^ For sham rats, craniotomy was performed without delivering an impact, serving as a surgical control to assess the effects of the procedure itself on circulating sEVs levels. Blood samples were collected at specific time points post‐injury to capture both acute changes immediately after injury, and chronic alterations in circulating sEVs levels over time. Utilizing this approach, we investigated whether our SERS‐based nanopillar chip allowed specific detection of ATP1B2‐ and EAAT2‐positive sEVs in circulation. We performed SEC on rat plasma to separate sEVs from free proteins and captured ATP1B2 and EAAT2 positive sEVs on our nanopillar chip. To quantify the number of brain specific sEVs and neuroinflammation, we measured the number of sEVs positive for ATP1B2, EAAT2, and the cytokine CCL2. We observed significant increases in circulating sEVs positive for ATP1B2 and EAAT2 after CCI injury that were sustained for 60 days post‐injury (**Figure** **4**). This suggests not only acute changes but also chronic alterations in sEVs release from the brain in response to a TBI. We also observed chronic TBI induced neuroinflammation with a sustained elevation of CCL2 positive sEVs from the brain post CCI injury compared to sham controls. Severe TBI often results in prolonged neuroinflammation,^[^ ^15^ ^]^ marked by the sustained activation of microglia and astrocytes. This persistent inflammatory state is associated with the release of pro‐inflammatory cytokines, such as CCL2, which can exacerbate neuronal damage and potentially drive chronic neurodegenerative processes following TBI. Our platform offers a novel and efficient approach to monitor these inflammatory changes, providing critical insights into the progression of neuroinflammation and its impact on recovery over time.

### 3.5 Profiling Brain sEVs Abundance and Neuroinflammation in Human Clinical Samples

**Figure 5:**
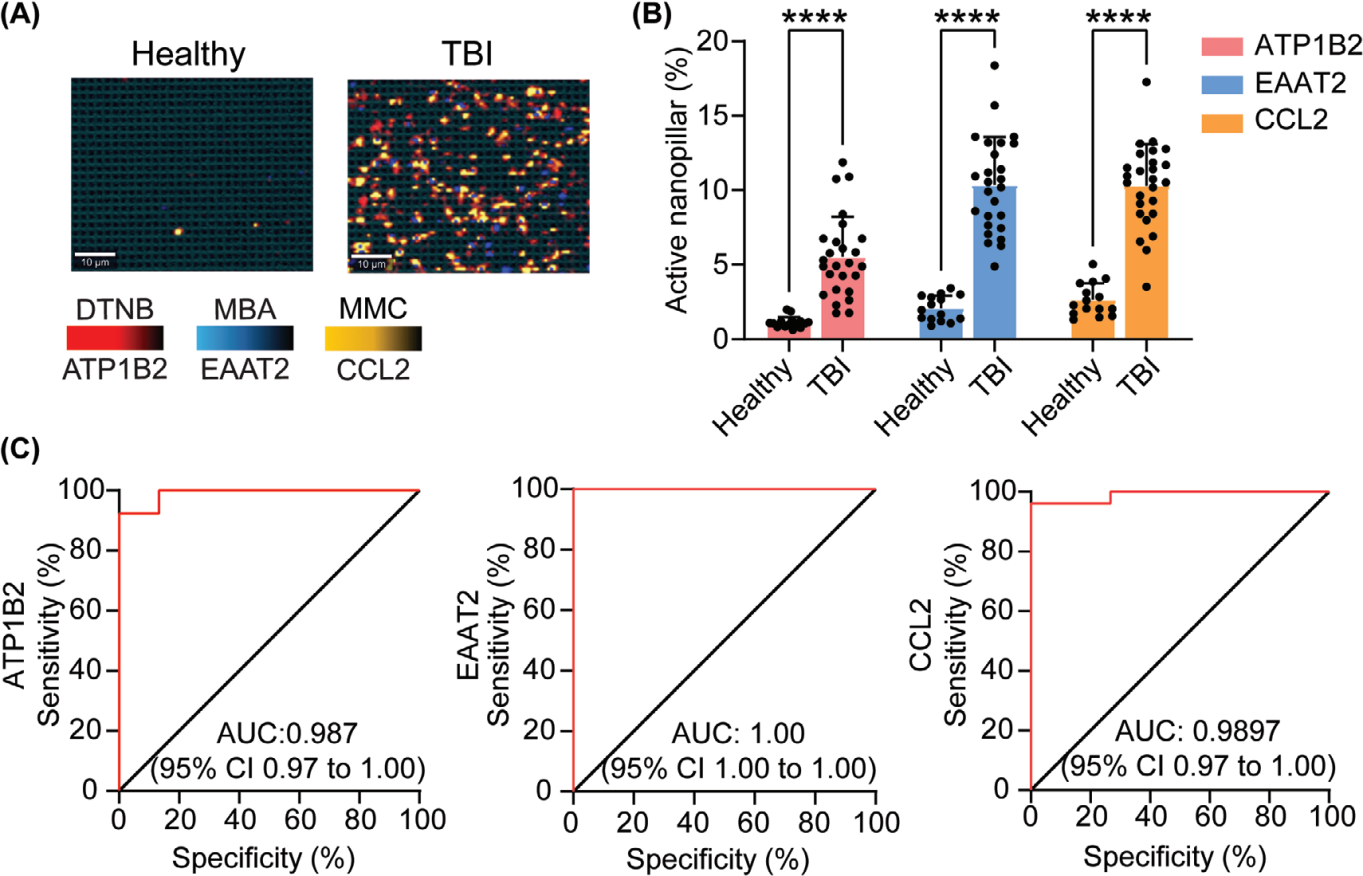
Analysis of brain sEVs signature in healthy individuals and in patients with TBI. A) Representative SERS mapping images of the brain‐specific sEVs from TBI patients compared to healthy controls. B) Corresponding sEV profiles from panel A demonstrating elevated ATP1B2, EAAT2, and CCL2 in patients with a TBI. *n* = 15 (healthy) *n* = 26 (TBI), data are represented as mean ± SD. ^****^ *p* < 0.001. C) ROC curve analysis for each marker between healthy and TBI patients.

To determine the diagnostic capability of our nanopillar chip for detecting a TBI, we investigated the accuracy of our approach in distinguishing brain specific sEVs isolated from the plasma of healthy individuals (*n* = 15) and TBI patients as determined by the GCS score (*n* = 26). Total circulating sEVs derived from patients with a TBI exhibited significant elevation of brain specific sEVs compared to healthy controls (**Figure** **5**A,B). Furthermore, brain‐specific sEVs in patients with a TBI had significantly elevated levels of CCL2, supporting the notion that we can directly profile inflammation originating in the brain (Figure 5A,B), and not generalized inflammatory responses from infection or other injuries. Receiver operating characteristic (ROC) curves were used to evaluate the diagnostic capability of our chip for detecting TBI. As shown in Figure 5C, ATP1B2, EAAT2, and CCL2 had area under the ROC curve (AUC) values of 0.987, 1, 0.9897, respectively, reflecting a highly accurate diagnostic capability. These findings demonstrated that our nanopillar chip can identify TBI patients and neuroinflammation with a high degree of accuracy by profiling brain‐specific sEVs. Although in a CCI model we demonstrated the longitudinal course of TBI‐induced neuroinflammation, future work tracking neuroinflammatory recovery in clinical samples will be essential for extending the translational value of sEV‐based biomarkers.

## 4 Conclusion

Our study presents a methodology for profiling single brain‐specific sEVs, offering a new window into measuring the brain's response to injury and inflammation. The SERS‐based nanopillar chip enables highly sensitive detection of low‐abundance, tissue‐specific sEV–cytokine signatures, providing a non‐invasive window into neuroinflammatory processes. While the platform is cost‐effective, clinical translation will require further optimization. In particular, an integrated optical measurement system is essential to support the implementation of SERS‐based diagnostics in clinical laboratory settings.^[^ ^38^ ^]^ In addition, although healthy individuals served as controls in this study, the absence of patients with systemic inflammation but no brain injury limits our ability to fully assess the specificity of sEV‐associated CCL2 for neuroinflammation. Validation in larger, longitudinal cohorts with varying degrees of severity will be necessary to establish clinical workflows, assess scalability, and evaluate the utility of sEV profiling in routine patient monitoring. We believe that the high sensitivity of the nanopillar chip, along with its ability to detect brain‐specific cytokine‐EVs in blood, will have implications for the development of brain‐specific biomarker diagnostics in TBI, as well as in other common forms of brain injury such as stroke, hypoxia, dementia, and other neurological disorders associated with inflammatory processes.

## Conflict of Interest

The authors declare no conflict of interest.

## Author Contributions

Z.Z. and R.J.L. contributed equally to this work. Z.Z. and R.J.L., designed and performed experiments, analyzed and interpreted the data, and directed the research. X.N. was involved in sEVs characterization. X.V.T. and F.N. provided clinical samples and participated in clinical data analysis. G.P. generated the brain organoids used for in vitro sEV analysis. C.W. Initiated and collected samples, proof‐read and revised the manuscript. Z.Z., R.J.L., R.L., X.V.T., F.N., and M.T. conceived the study and initiated the research. All authors discussed the results and participated in writing and revising the manuscript and approved the submitted version.

## Supporting information



Supporting Information

## Data Availability

The data that support the findings of this study are available from the corresponding author upon reasonable request.
