# Supplementary material for: Single Extracellular Vesicle Profiling to Define Brain Specific Traumatic Brain Injury Induced Neuro‐Inflammation
Source: Small Methods. 2025 May 19;9(7):2401931. doi: 10.1002/smtd.202401931 (PMC12285632; doi:10.1002/smtd.202401931)
Supplement: Supplementary file 1 — Supporting Information [file SMTD-9-2401931-s001.pdf]

small methods

Supporting Information

for *Small Methods*, DOI 10.1002/smtd.202401931

Single Extracellular Vesicle Profiling to Define Brain Specific Traumatic Brain Injury Induced Neuro-Inflammation

Zhen Zhang, Richard J Lobb, Rebecca E Lane, Xuan Vinh To, Xueming Niu, Fiach Antaw, Giovanni Pietrogrande, Craig Winter, Alain Wuethrich, Fatima Nasrallah* and Matt Trau**

Supplementary information

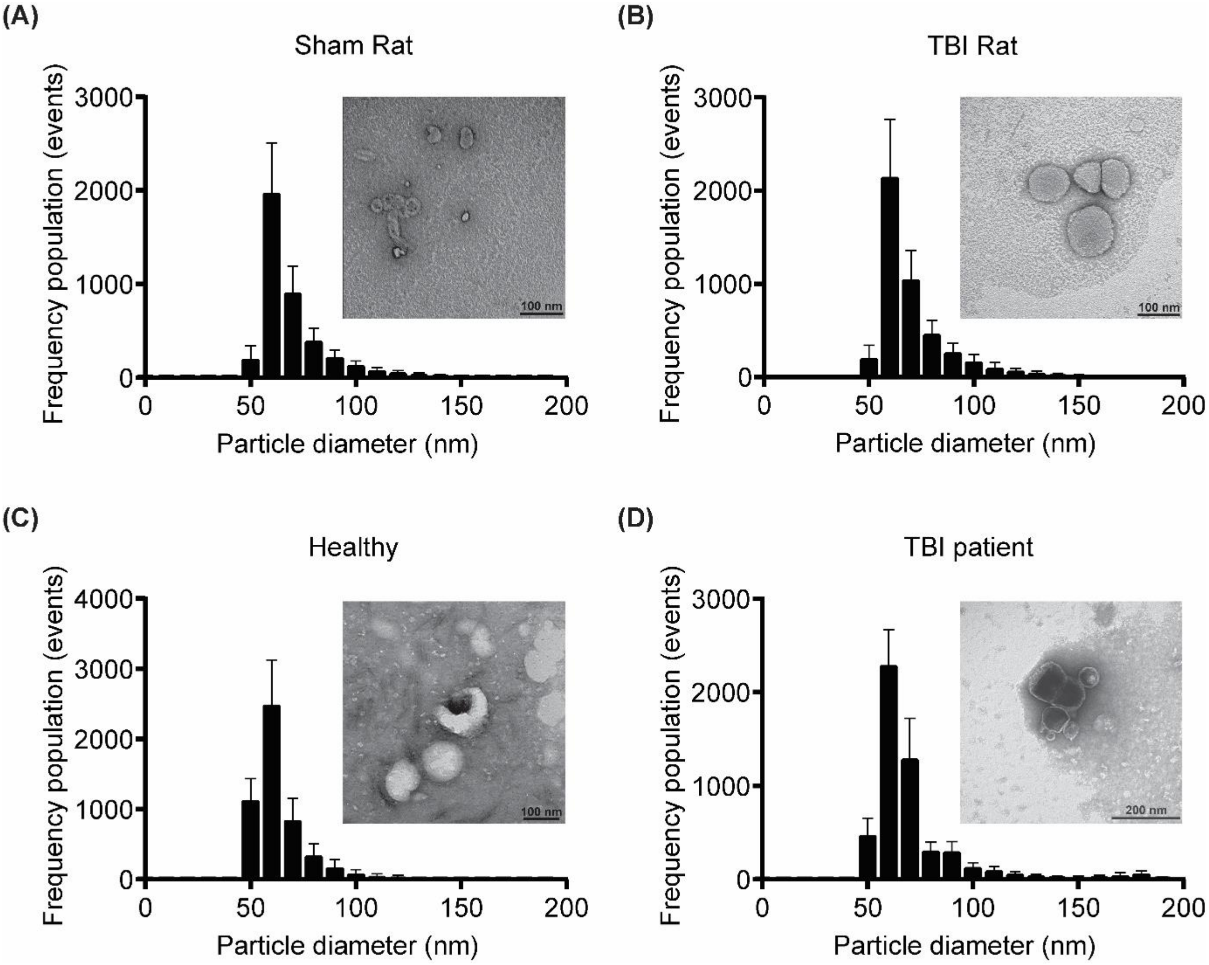

Figure S1. EV size characterization by nanoFCM and TEM. **(A)** Sham rat sEV size. Scale bar = 100 nm. **(B)** TBI rat sEV size. Scale bar = 100 nm. **(C)** Healthy individual sEV size. Scale bar = 100 nm. **(D)** TBI patient sEV size. Scale bar = 200 nm.

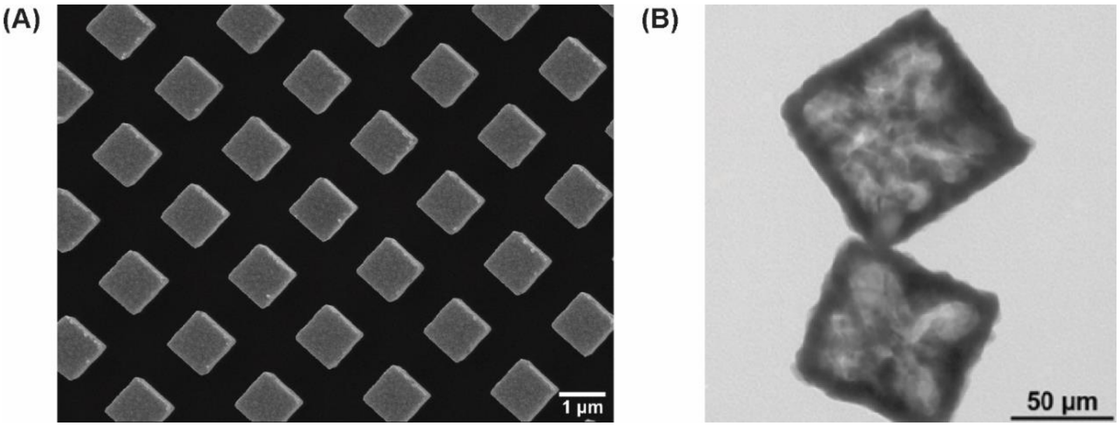

Figure S2. (A) Nanopillar integrated device characterization by scanning electron microscope (SEM). Scale bar = 1 μm. **(B)** Nanobox characterization by transmission electron microscopy (TEM). Scale bar = 50 μm.

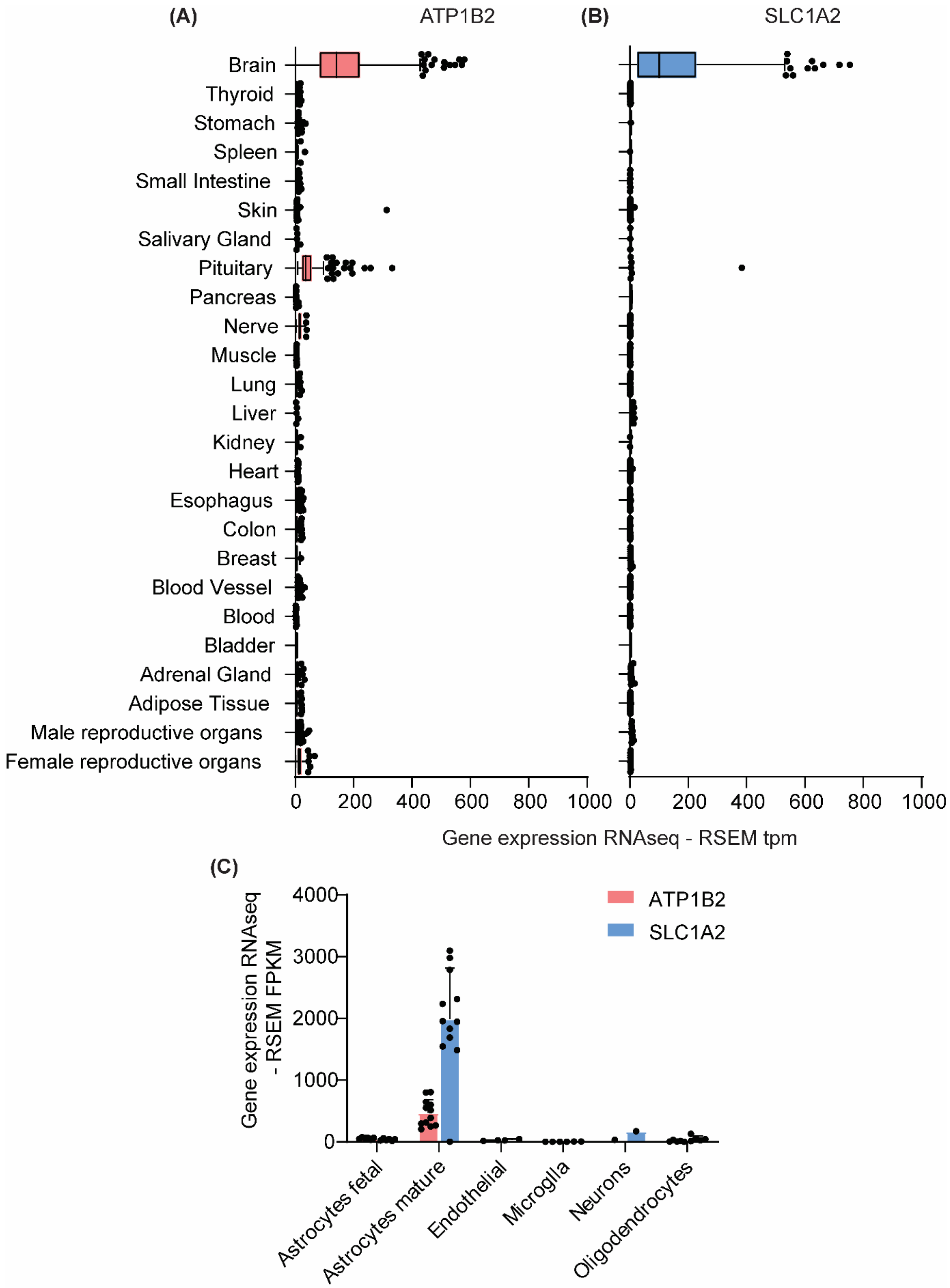

Figure S3. RNA expression of ATP1B2 **(A)** and SCL1A2 **(B)** from the GTEx database, and **(C)** the Brain-RNA-seq database. High expression levels of ATP1B2 and SCL1A2 are observed in brain tissue and mature astrocytes.

Supplementary table

Table S1. Clinical sample information

Characteristics	TBI	Healthy
Total sample	26	15
**Age**		
Median	37.5	28
Range	18-67	23-30
**Gender**		
F	5 (20%)	10 (67%)
M	21 (80%)	5 (33%)

Table S2. TBI patients' clinical information

Patient	Gender	Age at Injury	GSC Score	Injury Severity
Patient 1	M	40	13	Mild
Patient 2	M	23	13	Mild
Patient 3	M	54	13	Mild
Patient 4	F	67	15	Mild
Patient 5	M	52	15	Mild
Patient 6	F	34	14	Mild
Patient 7	F	N/A	13	Mild
Patient 8	M	N/A	15	Mild
Patient 9	M	N/A	9	Moderate
Patient 10	M	N/A	15	Mild
Patient 11	M	52	14	Mild
Patient 12	M	25	14	Mild
Patient 13	F	66	14	Mild
Patient 14	M	23	3	Severe
Patient 15	M	35	10	Moderate
Patient 16	M	19	7	Severe
Patient 17	M	23	12	Mild
Patient 15	M	48	12	Moderate
Patient 16	M	26	15	Mild
Patient 17	M	58	14	Mild
Patient 18	M	53	5	Severe
Patient 19	M	20	14	Mild
Patient 20	M	18	10	Moderate
Patient 21	M	20	15	Mild
Patient 22	M	50	15	Mild
Patient 23	F	41	14	Mild
Patient 24	M	40	13	Mild
Patient 25	M	23	13	Mild
Patient 26	M	54	13	Mild
